# Ferromagnetism from non-magnetic ions: Ag-doped ZnO

**DOI:** 10.1038/s41598-019-56568-8

**Published:** 2019-12-27

**Authors:** Nasir Ali, Vijaya A. R., Zaheer Ahmed Khan, Kartick Tarafder, Anuvesh Kumar, Manoj K. Wadhwa, Budhi Singh, Subhasis Ghosh

**Affiliations:** 1 School of Physical Sciences, Jawaharlal Nehru University, New Delhi, 110067 India; 2 Department of Physics, National Institute of Technology Karnataka, Surathkal, 575025 India; 3 Semi-Conductor Laboratory (SCL), Department of Space, Govt. of India, Sector 72, S.A.S Nagar, Punjab, 160071 India; 4 Inter University Accelerator Center, Aruna Asaf Ali Marg, New Delhi, 110067 India

**Keywords:** Materials science, Magnetic properties and materials

## Abstract

To develop suitable ferromagnetic oxides with Curie temperature (*T*_*C*_) at or above room temperature for spintronic applications, a great deal of research in doping ZnO with magnetic ions is being carried out over last decade. As the experimental results on magnetic ions doped ZnO are highly confused and controversial, we have investigated ferromagnetism in non-magnetic ion, Ag, doped ZnO. When Ag replaces Zn in ZnO, it adopts 4d^9^ configuration for Ag^2+^ which has single unpaired spin and suitable exchange interaction among these spins gives rise to ferromagnetism in ZnO with above room temperature *T*_*C*_. Experimentally, we have observed room temperature ferromagnetism (RTFM) in Ag-doped ZnO with Ag concentration varied from 0.03% to 10.0%. It is shown that zinc vacancy (*V*_*Zn*_) enhances the ferromagnetic ordering (FMO) while oxygen vacancy (*V*_*O*_) retards the ferromagnetism in Ag-doped ZnO. Furthermore, the theoretical investigation revealed that *V*_*Zn*_ along with Ag^2+^ ions play a pivotal role for RTFM in Ag-doped ZnO. The Ag^2+^-Ag^2+^ interaction is ferromagnetic in the same Zn plane whereas anti-ferromagnetic in different Zn planes. The presence of *V*_*Zn*_ changes the anti-ferromagnetic to ferromagnetic state with a magnetic coupling energy of 37 meV. Finally, it has been established that the overlapping of bound magnetic polarons is responsible for RTFM in low doping concentration. However, anti-ferromagnetic coupling sets in at higher doping concentrations and hence weakens the FMO to a large extent.

## Introduction

The diluted magnetic oxides (DMOs) and diluted magnetic semiconductors (DMSs) are important candidates for spintronics^1,2^. The ferromagnetism in DMOs and DMSs is highly debatable problem in contemporary condensed matter physics^3–7^. For a device application, the ferromagnetism in DMOs and DMSs must be intrinsic and Curie temperature (${T}_{C}$) should be above room temperature. The discovery of ferromagnetism in Mn-doped GaN^8^, ZnO^9^ and GaAs^2^ has led to search of most appropriate materials for spintronic devices operating at 300 K. Out of a variety of host materials for DMOs, ZnO has been extensively studied, after the theoretical prediction by Dietl *et al*.^4^ on the possibility of ${T}_{C}$ higher than room temperature in ZnO. Subsequently, there are several reports that claimed RTFM in Mn^9^, Co^10^, Ni^11^ and Fe^12^ doped ZnO. However, the origin and exchange mechanism responsible for RTFM in these systems are still controversial. Although, several mechanisms based on different kind of exchange interactions have been presented to explain RTFM in magnetic ions doped ZnO^13–18^, extrinsic origin of RTFM has not also been ruled out^19–21^. Whatever be the exchange mechanisms of ferromagnetism, one has to explain the most important experimental facts in ZnO *i*.*e*. the observation of ferromagnetic ordering (FMO) at 300 K at a very low doping content (0.03%) which is several order of magnitude smaller than the cation percolation threshold (${x}_{p}$), ~19.8% in wurtzite ZnO^22^. This indicates that all the short range interaction such as double and super exchange proposed so far^23,24^, except the mechanism proposed by Coey *et al*.^14^, to explain FMO can be doubted as the distance (~20 nm) between two dopant atoms is much larger than the lattice constant of ZnO. This necessitates the role of third party in the mechanism for RTFM. As both oxygen vacancy (${V}_{O}$) and zinc vacancies (${V}_{Zn}$) are always abundant in ZnO grown under any conditions^25–29^, these intrinsic defects could facilitate interaction between distantly localized spins^30–36^. To rule out the doubt whether ferromagnetism is intrinsic or extrinsic in DMOs, it is now being attempted to dope ZnO with non-magnetic ions. Ag is the most suitable candidate for this investigation because (i) the substitution of Ag to Zn sites have 4d^9^ outer shell configuration with one unpaired spin, (ii) neither metallic Ag and nor its clusters are ferromagnetic and (iii) AgO and Ag_2_O are not ferromagnetic. There are conflicting reports on RTFM in Ag-doped ZnO. Ma *et al*.^37^ have found no ferromagnetic ordering in Ag-doped ZnO whereas the experimental report in ref. ^38^ have provided evidence for RTFM in Ag-doped ZnO. The RTFM observed so far in Ag-doped ZnO is very weak and unstable raising a doubt whether RTFM is due to Ag^2+^ (4d^9^) or due to intrinsic defects. Deng *et al*.^39^ observed RTFM in Ag-doped ZnO nanowires and nanocrystals when Ag nanocrystals are caped with thiol molecule. In view of all these contradictory results regarding the stability and origin of RTFM in Ag-doped ZnO, we present a detailed experimental and theoretical investigation to understand the origin of RTFM in Ag-doped ZnO films. Furthermore, there is plenty of works on 3d magnetic and non-magnetic ions doped wide gap oxides, but there are very few works on magnetization in 4d ion doped ZnO or other oxide semiconductors.

## Experimental Details

The radio frequency (RF) magnetron sputtering was used to grow Ag-doped ZnO films on transparent quartz and silicon substrates at an optimized growth temperature of 600 °C^40–43^. ZnO (99.999%) and AgO (99.999%) (Sigma Aldrich, USA) powders were mixed together, grounded for fifteen to twenty hours and sintered at 800 °C for 20 hours to synthesize sputtering targets by standard solid state reaction. We have used Ar and O_2_ as a sputtering and reacting gases. The Ar and O_2_ partial pressure were varied to grow 2% Ag-doped ZnO films under excess and deficient oxygen atmosphere. The thicknesses of the doped and undoped ZnO films are determined by using SOPRA GES5E spectroscopic ellipsometer. Magnetic measurements on Ag-doped ZnO films were carried out by superconducting quantum interference device (EverCool MPMS XL-7, USA). X-ray diffraction (XRD) of Ag-doped ZnO films were carried out by PANalytical X’pert Pro system with nickel filtered Cu *K*_*α*_ X-ray source. Bruker model EMX MicroX system was used to perform electron paramagnetic resonance (EPR) on doped ZnO films. Photoluminescence (PL) measurements were carried out with Ar ion laser with operating wavelength of 353 nm. The chemical analysis of Ag in ZnO with oxidation state has been carried out by X-ray photoelectron spectroscopy (XPS) (Omicron ESCA, Oxford Instrument Germany). The Rutherford backscattering spectrometry (RBS) measurements were performed in a vacuum of 10^−6^ mbar using a 2.0 MeV He^+^ ion beam in a General Ionex Tandetron accelerator. RBS spectra were analyzed using the Rutherford universal manipulation programme (RUMP), a computer simulation program. Temperature dependence of rsesistivity was measured using Keithley 2611A sourcemeter unit and closed cycle He refrigerator. Keithley 2182A nanovoltmeter and 6221 AC and DC current source were used to perform room temperature Hall measurements on doped ZnO films.

## Results

### Structural and compositional analysis

**Figure 1:**
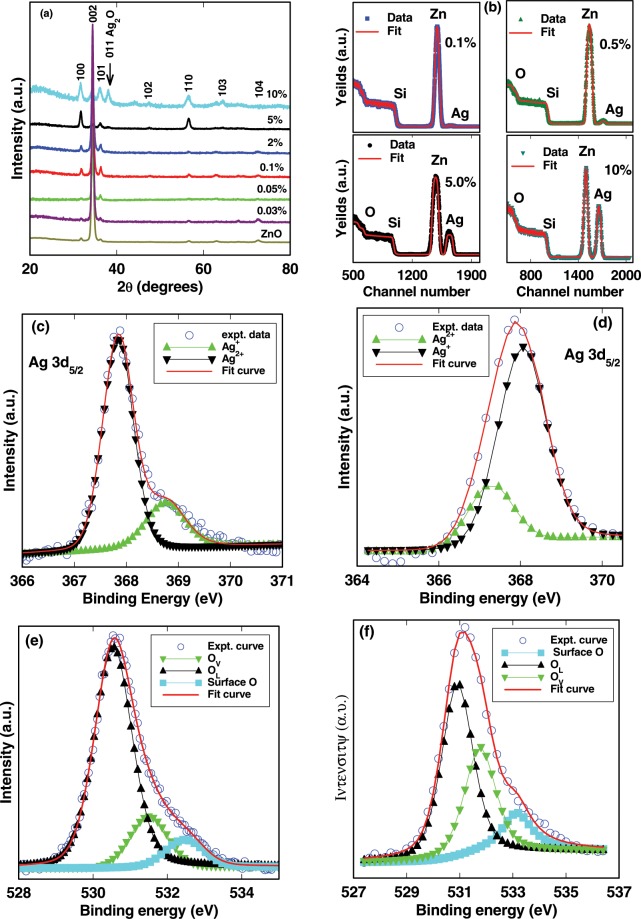
(**a**) XRD spectra of undoped and different Ag contents doped ZnO films. Arrow indicates the position of Ag_2_O phase in 2% and 5% Ag-doped ZnO films. (**b**) RBS spectra of different Ag contents doped ZnO films. The solid red lines show simulation using RUMP. XPS data of 3*d*_5/2_ of (**c**) 2% and (**d**) 5% Ag-doped ZnO films. The open circle shows the experimental data of 2% and 5% Ag-doped ZnO films. The thick solid red line which is sum of two peaks fitted with Gaussian due to ${{\rm{Ag}}}^{1+}$ and ${{\rm{Ag}}}^{2+}$ charge states of 2% and 5% Ag-doped ZnO films, is fit to experimental data. High resolution O 1*s* XPS spectra of (**e**) 2% and (**f**) 5% Ag-doped ZnO films. The open circle shows the experimental data of 2% and 5% Ag-doped ZnO films. The thick solid red line which is sum of three peaks fitted with Gaussian due to lattice oxygen (${O}_{L}$), deficiency of oxygen (${O}_{V}$) and absorbed or dissociated oxygen on the surface of 2% and 5% Ag-doped ZnO films, is fit to experimental data.

In order to study the presence of different phases of Ag in Ag-doped ZnO films, a careful XRD analysis has been carried out. Figure 1a shows the XRD spectra of undoped and different Ag-doped ZnO films. A common (002) peak at 34.4° indicates that Ag-doped ZnO films up to 2% doping concentration are highly c-axis oriented and monophasic in nature with no observed peaks from the secondary phase of Ag. The Ag-doped ZnO films with higher Ag concentration of 5% and 10% show another peak at ~38° due to Ag_2_O (011). The large ionic radius of Ag^1+^ (128 pm) as compare to Zn^2+^ (74 pm) is probably responsible for secondary phase formation. Figure 1a also shows that the ZnO doped with more than 2% Ag are not c-axis oriented. Ag_2_O (011) peak and other non c-axis oriented peaks, (100), (101),(102),(110) (103) are observed. Ag_2_O phase drives the c-axis oriented films to misoriented Ag-doped ZnO films. He *et al*.^38^ observed similar results in Ag-doped ZnO. But, it is noticed that there is no shift in the (002) peak with Ag content, implying that internal chemical pressure due to difference in ionic sizes of Zn^2+^ (0.074 nm) and Ag^2+^ (0.108 nm) ion has little or no effect on the crystal structure of host ZnO. The RBS spectra of different Ag-doped ZnO films have been shown in Fig. 1b. The Ag-doped ZnO films with low thicknesses were used to resolve Zn and Ag peaks. The RBS spectra of different Ag-doped ZnO films revealed that the intensity of the Ag peak increases with Ag contents. This suggests that Ag ions go into the ZnO matrix. Figure 1b also shows the typical simulation using RUMP^44^ which resulted similar dopants concentration in films as stiochiometrically intended in sputtering targets. The thicknesses of all Ag-doped ZnO films obtained from RBS simulation match with values obtained from SE. All the parameters simulated by the RUMP are summarized in Table 1.

**Table 1:** Simulated parameters obtained from RUMP programme for ZnO thin films doped with different Ag content.

Zn_1−*x*_Ag_*x*_O	Thickness (nm) (RBS)	Thickness (nm) (SE)	Zn (conc.)	O (conc.)	Ag (conc.)
$x=0$	80	75	0.5	0.5	0
$x=0.001$	90	80	0.4995	0.4995	0.001
$x=0.005$	125	115	0.4975	0.4975	0.005
$x=0.01$	200	210	0.495	0.495	0.01
$x=0.05$	145	150	0.475	0.475	0.05
$x=0.1$	90	80	0.45	0.45	0.1

XPS was used to investigate the bonding characteristics and oxidation states of Ag in Ag-doped ZnO films. A detailed survey scan and high resolution scans of Zn 2*p*, Ag 3*d* and O 1*s* peaks were performed. C 1*s* line was used for calibration. The high resolution scan of Ag 3*d* XPS peaks obtained from the 2% and 5% Ag-doped ZnO films are shown in Fig. 1c,d, respectively. Figure 1c,d also show that the XPS peaks can be fitted with contribution from two charge states of Ag. The 3*d*_5/2_ peak is deconvoluted by fitting which results mixed oxidation states (+1 and+2) of Ag at 367.78 eV and 367.85 eV in 2% and 5% Ag-doped ZnO, respectively. These binding energy values are in good agreement with published XPS data for AgO and Ag_2_O^45,46^. Park *et al*.^47^ calculated the electronic band structure for AgO and concluded that the two Ag ions are in +1 and +2 charge states due to the presence of holes on the oxygen species. The mixed oxidation (1+ and 2+) states of Ag in ZnO indicate that in addition to substitutional sites, substantial numbers of Ag atoms occupy interstitial sites in both 2% and 5% Ag-doped ZnO films. The ratio of 2+ oxidation state to 1+ oxidation state of Ag are ~3:1 and ~1:3 in 2% and 5% Ag-doped ZnO films, respectively. This indicates that number of Ag atoms occupy the interstitial sites increase with Ag doping concentration. The +1 charge state of Ag in heavily doped ZnO with concentrations of Ag > 2% (5% and 10%) can also come from Ag_2_O, as shown in XRD results. Figure 1e,f show the high-resolution O 1*s* XPS peak for 2% and 5% Ag-doped ZnO films, respectively. The fitted peaks are centered at 530.54 eV, 531.5 eV and 532.46 eV for 2% Ag-doped ZnO and 530.91 eV, 531.76 eV and 533.14 for 5% Ag-doped ZnO. The lower binding energy peaks are assigned to O^2−^ ions due to Zn-O bonding of the wurtzite structure of ZnO whereas medium binding energy peaks are attributed to deficiency of oxygen in hexagonal wurtzite ZnO^48,49^. The higher binding energy peaks are assigned to the absorbed or dissociated oxygen on the surface^48,49^. The area under of peak due to *V*_*O*_ for 2% and 5% Ag-doped ZnO are 3050.37 and 4188.84, respectively.

### Defects study

**Figure 2:**
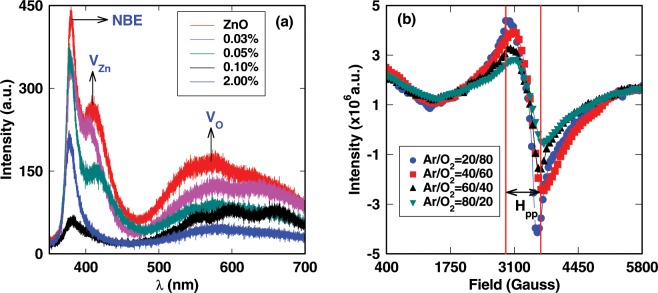
(**a**) PL spectra of undoped and different Ag contents doped ZnO films at 300 K. NBE denotes the near band edge emission due to bound exciton. (**b**) EPR of 2% Ag-doped ZnO in different Ar/O_2_ ratio at room temperature.

As discussed before, to explain the RTFM in very lightly doped (~0.03%) ZnO, it is important to include the role of intrinsic defects which are always present in plenty in ZnO grown under any conditions (*i*.*e*. O-rich or Zn-rich condition). The authors in refs. ^30–32^ have emphasized the role of intrinsic defects on RTFM in different oxides doped with magnetic ions. The PL spectra of undoped and doped ZnO with different Ag content are shown in Fig. 2a. The three distinct PL peaks at 380 nm, 410 nm and 570 nm have been observed in undoped and Ag-doped ZnO films. The first PL peak at 380 nm can be attributed to near band edge transition due to bound exciton^50^. Based on energetic position, the peak at 410 nm can be assigned to the transition from ${V}_{Zn}$ levels to conduction band. Similar assignment of the PL peaks are reported in refs. ^51^ and^52^. The broad peak at 570 nm in visible region is due to ${V}_{O}$ related defects in undoped and Ag-doped ZnO films^50^. It can emphasized that PL spectra are the signature of abundant intrinsic defects in undoped and doped ZnO. The room temperature EPR spectra of 2% Ag-doped ZnO films grown at different Ar/O_2_ ratio confirmed the presence of native defects (Fig. 2b). The peak in EPR spectra of 2% Ag-doped ZnO corresponds to g = 2.013 and can be assigned to ${V}_{Zn}$. Similar values of g, g = 2.0127 and g = 2.0155 have been attributed to ${V}_{Zn}$ in refs. ^53^ and^54^, respectively. Taylor *et al*.^55^ also observed the ${V}_{Zn}$ in ZnO with g value ranging from g = 2.0018 to g = 2.054. This indicates that the ${V}_{Zn}$ is a dominant defect in 2% Ag-doped ZnO films. It has been observed that the intensity of EPR peak increases when Ag-doped ZnO films grown under oxygen-rich atmosphere (having more ${V}_{Zn}$). The number of EPR active spins associated with ${V}_{Zn}$ can be approximately expressed by relation, $N\propto {\rm{A}}\,{(\Delta {H}_{pp})}^{2}$, where A is the amplitude, $N$ is the concentration of spins, and $\Delta {H}_{pp}$ is the peak to peak width of the first derivative of EPR spectrum^56^. Using this relation, concentration of ${V}_{Zn}$ has been found to be order of 10^18^ cm^−3^.

### Electrical transport

**Figure 3:**
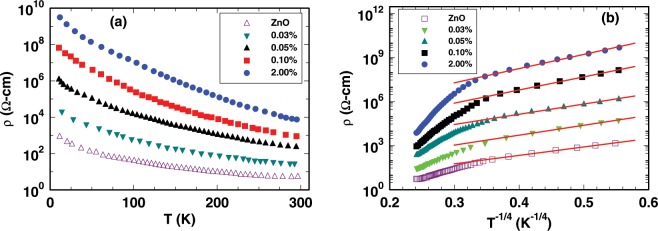
(**a**) Electrical resistivity versus temperature of undoped and different Ag contents doped ZnO films. (**b**) The logarithm of resistivity $\rho$ versus ${(1/T)}^{1/4}$ of undoped ZnO and different Ag contents doped ZnO films. The red solid straight lines are the fit with the Mott 3D VRH law in Eq. 1, the values of fitting parameters are listed in Table 2.

The temperature dependence of resistivity of undoped and Ag-doped ZnO films are shown in Fig. 3a. In all samples, the resistivity decreases with temperature, suggesting the typical semiconducting behavior of undoped and doped ZnO. It has been observed that resistivity increases with Ag contents in Ag-doped ZnO. This is further corroborated by the room temperature Hall measurements which indicate that the Ag doping suppressed the carrier concentration in Ag-doped ZnO by more than four orders of magnitude (Table 2). Figure 3b shows the linear relationship between *ln*$\rho$ and *T*^−1/4^ in the temperature range from 10 K to 90 K, suggesting that the Mott variable range hopping (VRH) dominates the electrical transport in both doped and undoped ZnO thin films. However, in the temperature range from 90 K to 300 K, the deviation of the resistivity from Mott VRH conduction indicates that thermal activation of carrier takes place in undoped and Ag-doped ZnO films above 90 K. Mott’s VRH conduction in 3-dimensions is given by^57–59^,

1 $$\rho (T)={\rho }_{M}\,exp[{(\frac{{T}_{M}}{T})}^{\mathrm{1/4}}]$$

**Table 2:** Electrical transport fitting parameters extracted from Mott VRH at 80 K and and room temperature carrier concentrations (n) were determined from Hall effect measurement.

Zn_1−*x*_Ag_*x*_O	*T*_*M*_ (10^4^ *K*)	*R*_*hop*,*Mott*_ (nm)	*W*_*hop*,*Mott*_ (meV)	n(cm^−3^) (10^18^)	$\tfrac{{{\boldsymbol{R}}}_{{\boldsymbol{hop}},{\boldsymbol{Mott}}}}{{\boldsymbol{\xi }}}$
$x=0$	3.0	2.5	17.9	1.13	1.2
$x=0.0003$	5.9	2.9	21.2	0.32	1.4
$x=0.0005$	6.5	3.0	21.8	0.04	1.5
$x=0.001$	18.0	3.9	27.9	0.0012	1.9
$x=0.02$	24.7	4.2	30.3	0.0007	2.1

The characteristics parameters ${\rho }_{M}$ and *T*_*M*_ are defined as

2 $${\rho }_{M}={\mathrm{(3}{e}^{3}{\nu }_{ph})}^{-1}{(\frac{N({E}_{F})}{8\pi \alpha {k}_{B}})}^{-\mathrm{1/2}}$$

and

3 $${T}_{M}=\frac{\lambda }{{\xi }^{3}{k}_{B}N({E}_{F})},$$

where *k*_*B*_, $N({E}_{F})$, ${\nu }_{ph}$, $\xi$, $\alpha$ and $\lambda$ are Boltzmann constant, electronic density of states at Fermi level, phonon frequency at Debye temperature (=10^13^ *s*^−1^), electronic localization length, inverse of localization length and dimensionless constant (=16)^60^, respectively. The hopping distance and hopping energy are

4 $${R}_{hop,Mott}=\frac{3}{8}\xi {(\frac{{T}_{M}}{T})}^{1/4}$$

and

5 $${W}_{hop,Mott}=\frac{1}{4}{k}_{B}T{(\frac{{T}_{M}}{T})}^{1/4},$$

respectively. Localization length is equal to the effective Bohr radius, *a*_*B*_ (2 nm) of shallow donors in ZnO^61^. We have calculated the values of ${R}_{hop,Mott}$ and ${W}_{hop,Mott}$ in temperature range from 10 K to 90 K using the experimental values of ${\rho }_{M}$ and ${T}_{M}$ and listed in Table 2. Increase in the hopping energy and hopping distance with Ag contents in ZnO, indicates that a large number of carriers become strongly localized at higher doping concentration. The conditions for VRH conduction are (i) the value of $\frac{{R}_{hop,Mott}}{\xi }$ must be greater than 1, and (ii) ${W}_{hop,Mott}$ must be greater than ${k}_{B}T$. Note that both of these conditions are satisfied in the undoped and Ag-doped ZnO films. Such strongly localized carriers are unable to mediate any magnetic exchange interaction in Ag-doped ZnO films. Hence, we can ruled out Ruderman– Kittel– Kasuya– Yosida exchange interaction in Ag-doped ZnO films.

### Magnetization study

**Figure 4:**
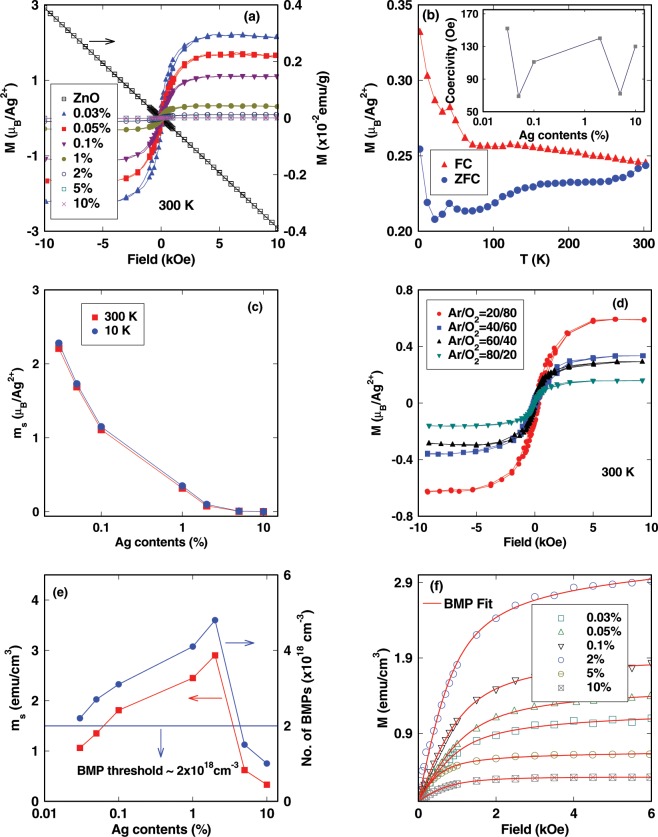
(**a**) Magnetization versus applied field curves of undoped and different Ag-doped ZnO films at 300 K. (**b**) M versus T for 2% Ag-doped ZnO film for both field cooled and zero field cooled. (**c**) Saturated magnetic moment per Ag^2+^ ion with different Ag contents measured at 300 K and 10 K. Connecting lines are guide for eyes. (**d**) Magnetization versus applied field curves of 2% Ag-doped ZnO films grown in different Ar and O_2_ partial pressure at 300 K. (**e**) Saturated magnetic moment per *cm*^3^ and BMPs concentration with different Ag contents at 300 K. Connecting lines are guide for eyes. Horizontal line indicates the percolation threshold for BMPs in the hexagonal wurtzite ZnO. (**f**) Initial portion of M-H curves of different Ag contents doped ZnO at 300 K, fitted with BMP model. Symbols are for experimental data and the solid red line is a fit with BMP model.

Figure 4a shows the magnetization result of undoped and Ag-doped ZnO films at 300 K. The bifurcation in zero field cooled (ZFC) and field cooled (FC) at 500 Oe of 2% Ag-doped ZnO shown in Fig. 4b indicate that the ${T}_{C}$ is above room temperature. The undoped ZnO film grown under optimized conditions is diamagnetic (Fig. 4a). It has been observed that Ag-doped ZnO films have a significant value of coercivities which are almost independent on doping concentration (inset of Fig. 4b). Figure 4c shows the variation in a saturated magnetic moment (${m}_{s}$), expressed as ${\mu }_{B}$ per ${{\rm{Ag}}}^{2+}$ with Ag content at 10 K and 300 K, indicating the temperature independent magnetization. The observed magnetic moment in Ag-doped films can be understood by the possible electronic configuration of Ag in ZnO. Ag atom in unionized states has an outer shell configuration of [Kr] ${{\rm{4d}}}^{10}\,{{\rm{5s}}}^{1}$. The +1 and +2 charge states of Ag have [Kr] $4{d}^{10}$ and [Kr] $4{d}^{9}$ configuration, respectively. In d^10^ configuration, all the d electrons are paired and hence ${{\rm{Ag}}}^{1+}$ ions in ZnO do not contribute to magnetic moment whereas ${{\rm{Ag}}}^{2+}$ ions in ZnO with d^9^ configuration have one unpaired electron which gives rise to a spin angular momentum of 1/2. Therefore, from the relation, $\mu =g{\mu }_{B}\sqrt{s(s+1)}$; g = 2, s = 1/2, the net magnetic moment should be 1.72 *μ*_*B*_ per ${{\rm{Ag}}}^{2+}$ ion which matches well with observed value of ${m}_{s}$ for 0.03% and 0.05% Ag-doped ZnO films at 300 K^40^. The ${m}_{s}$ decreases with Ag concentration in Ag-doped ZnO films (Fig. 4c). When doping concentration of Ag is high, the Ag atoms in the wurtzite lattice can occupy cation sites in different Zn planes which give rise to antiferromagnetically aligned spins and hence reduce ${m}_{s}$ to a large extent (will be discussed in the next section). This is corroborated by XPS analysis as discussed in previous section. The number of Ag^1+^ (Ag^2+^) increases (decreases) with Ag doping concentration, resulting the reduction of ${m}_{s}$ substantially in heavily doped ZnO. But, in case of samples doped with low doping concentration of Ag, ferromagnetism strongly depends on native defects as long rang FMO in ZnO with relatively large ${x}_{p}$ can only be achieved by the participation of native defects^23,30–36^. In order to demonstrate the role of native defects (${V}_{Zn}$ and ${V}_{O}$) on FMO in Ag-doped ZnO films, we have grown 2% Ag-doped ZnO films in different Ar and O_2_ partial pressure (Fig. 4d). The authors in refs. ^26,35,62^ suggested that the O-rich atmosphere creates more ${V}_{Zn}$ compared to the O-deficient atmosphere. Increase in ${m}_{s}$ per Ag^2+^ ion with O_2_ partial pressure indicates that ${V}_{Zn}$ enhance the FMO in Ag-doped ZnO films whereas ${V}_{O}$ retards the FMO in Ag-doped ZnO. This is further corroborated by XPS analysis of O 1*s* peak of 2% and 5% Ag-doped ZnO. The area under of peak due to ${V}_{O}$ increases with Ag concentration indicating that the ${V}_{O}$ quenches the ${m}_{s}$ to a large extent in Ag-doped ZnO. Figure 4e shows variation in *m*_*s*_ per cm^3^ with Ag content. Figure 4f shows the virgin curve of M-H loops of Ag-doped ZnO at 300 K fitted with native defect based bound magnetic polaron (BMP) model which is discussed in next section^63^.

### DFT calculations

**Figure 5:**
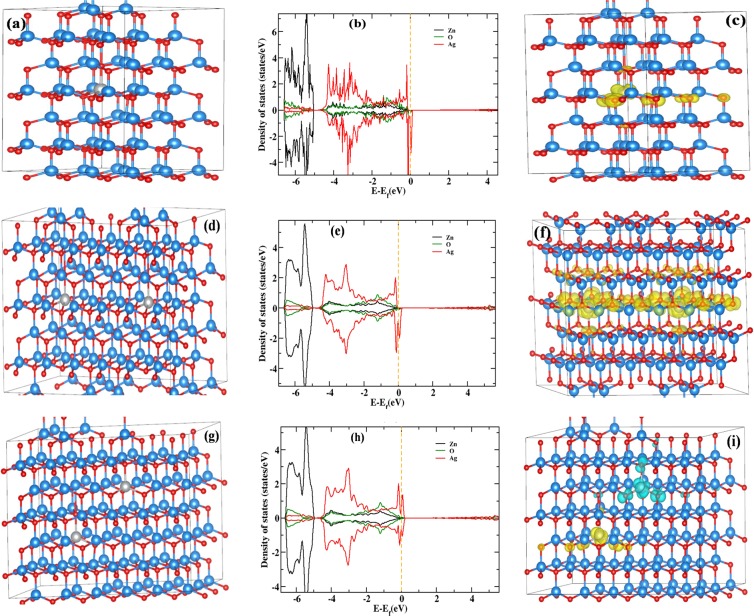
(**a**) Single unit cell with one doped Ag atom. (**b**) Partial DOS of Zn, O and Ag atoms in case (**a**). (**c**) Distribution of magnetization density of single unit cell with one Ag atom. (**d**) Two Zn atoms replaced by two Ag atoms in same Zn plane in super cell of 216 atoms (108 Zn and 108 O). (**e**) Partial DOS of Zn, O and Ag when two Ag atoms in same Zn plane (**d**). (**f**) Distribution of magnetization density over the same Zn plane. (**g**) Two Zn atoms replaced by two Ag atoms in different Zn planes in super cell of 216 atoms (108 Zn and 108 O). (**h**) Partial DOS of Zn, O and Ag when two Ag atoms in different Zn planes (**d**). (**i**) Distribution of magnetization density over the different Zn planes. There is almost no overlap between the magnetization densities. The spin-up and/or spin-down DOS is shown above and/or below the abscissa axis. The color code is same for all structure figure: light blue balls represents Zn-atom, red balls represent O atom and gray/magenta balls represent doped Ag atom. Yellow isosurface represents spin up (+Ve) magnetization density and light green isosurface represents spin down (−Ve) magnetization density. In the DOS plot: black line is for Zn, green line is for O and red line is for Ag projected density of states.

In order to understand the ferromagnetism in Ag-doped ZnO, a full potential Vienna *Ab initio* Simulation Package (VASP) was used to perform first principle density functional theory (DFT) calculations^64,65^ with (i) projector augmented wave (PAW) potentials^66,67^, (ii) expanded wave functions in the plane wave basis set with a kinetic energy cutoff of 500 eV and (iii) generalized gradient approximation (GGA) with Perdew– Burke– Ernzerhof (PBE) parameterization for exchange correlation functional^64^. The ZnO has the hexagonal wurtzite structure with optimized lattice parameter of a = 3.28 Å and c = 5.33 Å. The forces on each atom were calculated using the Hellmann-Feynman theorem and were subsequently used for conjugate gradient structural relaxation. Till the forces on the atom are converged to less than 0.01 eV/Å, the structure was optimized continuously. First of all, we made single unit cell of 54 Zn atoms. To test whether Ag as magnetic or nonmagnetic impurity in ZnO, we have replaced one Zn atom out of 54 Zn atoms by one Ag atom as shown in Fig. 5a. The partial density of states (DOS) of Ag, Zn and O have been shown in Fig. 5b. This suggest that Ag behaves as a magnetic impurity in the system whereas Zn and O show diamagnetic behavior. The net magnetic moment of the unit cell is 1 *μ*_*B*_. The magnetization density is localized near the Ag atom and small amount of magnetization density is also distributed on O atom which is located near Ag atom (Fig. 5c). To understand the magnetic interaction between the magnetic impurity center, we have performed super cell calculation. Here, we consider a large super cell of total 216 atoms (108 Zn and 108 O atoms). Out of 108 Zn atoms two are randomly chosen and replaced with two Ag atoms. There are two different ways for replacing two Zn atoms by two Ag atoms as (i) both the impurities are in the same Zn plane at a distance 9.79 Å, and (ii) impurities are in two different Zn planes at distance of 7.68 Å. Figure 5d shows the super cell with two Ag atoms separated by a distance of 9.79 Å. The partial DOS of Zn, Ag and O when two Ag atoms separated by 9.79 Å has been shown in Fig. 5e. The ferromagnetic state is more stable compared to anti-ferromagnetic state with a coupling energy of 44 meV. This suggest that ferromagnetic exchange interaction is a long ranged in Ag-doped ZnO as the distance between two Ag atoms is 9.79 Å. The magnetic moment on each Ag atom is 0.3 *μ*_*B*_ whereas the total magnetic moment of the unit cell is 3.91 *μ*_*B*_. Magnetization density is only distributed over the same Zn-plane (Fig. 5f). Figure 5g shows the super cell when two Ag atoms are in different Zn planes and separated by a distance of 7.68 Å. Figure 5h shows the partial DOS of Zn, Ag and O when two Ag atoms are in different Zn planes and separated by 7.68 Å. There is almost no overlap between the magnetization densities (Fig. 5i). Interestingly, a very weak anti-ferromagnetic coupling between two magnetic centers was observed in this case, with coupling energy of 21 meV. A super-exchange between two neighboring magnetic center through O atoms, could be responsible for this week anti-ferromagnetic coupling with a sufficiently low coupling energy.

**Figure 6:**
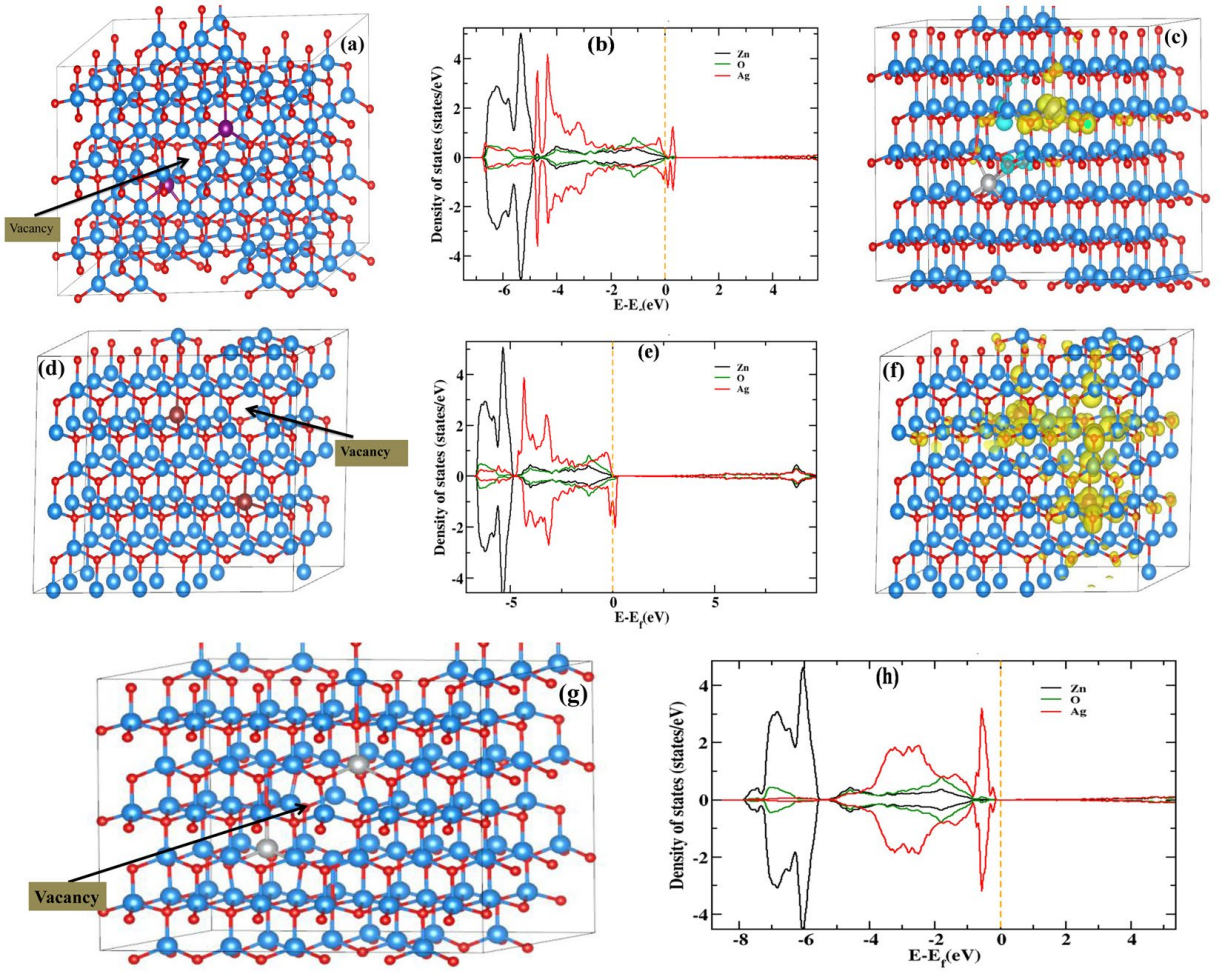
(**a**) Two Ag atoms in super cell of 216 atoms (108 Zn and 108 O) and ${V}_{Zn}$ lies between two Ag atoms. Arrow indicates the position of ${V}_{Zn}$ vacancy. (**b**) Partial DOS of Zn, O and Ag in case of (**a**). (**c**) Magnetization density when ${V}_{Zn}$ lies between two Ag atoms. (**d**) Two Ag atoms in super cell of 216 atoms (108 Zn and 108 O) and ${V}_{Zn}$ lies far from two Ag atoms. Arrow indicates the position of ${V}_{Zn}$ vacancy. (**e**) Partial DOS of Zn, O and Ag in case of (**d**). (**f**) A significant spin density was found the neighboring Zn and O atoms of the impurity and vacancy site. (**g**) Two Ag atoms in super cell of 216 atoms (108 Zn and 108 O) and ${V}_{O}$ lies between two Ag atoms. (**h**) Partial DOS of Zn, O and Ag in case of (**g**). The spin-up and/or spin-down DOS is shown above and/or below the abscissa axis. The color code is same for all structure figure: light blue balls represents Zn-atom, red balls represent O atom and gray/magenta balls represent doped Ag atom. Yellow isosurface represents spin up (+Ve) magnetization density and light green isosurface represents spin down (−Ve) magnetization density. In the DOS plot: black line is for Zn, green line is for O and red line is for Ag projected density of states.

In order to understand the role of ${V}_{O}$ and ${V}_{Zn}$ to ferromagnetism in Ag-doped ZnO, we have modified the structure by creating 1% of ${V}_{O}$ and ${V}_{Zn}$ vacancies. The ${V}_{Zn}$ has been introduced in the system by two different ways. In first case, ${V}_{Zn}$ lies between two doped Ag atoms and in another case ${V}_{Zn}$ lies far from the both Ag atoms. Figure 6a shows the super cell when ${V}_{Zn}$ lies between two Ag atoms. Figure 6b shows the partial DOS of Zn, O and Ag when ${V}_{Zn}$ lies between two Ag atoms. The magnetic moment of the Ag atom near the vacancy site quench down to zero (Fig. 6c). However, the other Ag atom behaves as an isolated magnetic center. The total magnetic moment on the isolated Ag atom and super cell are 0.3 *μ*_*B*_ and 0.8 *μ*_*B*_, respectively. Figure 6d shows the super cell when ${V}_{Zn}$ far from both the Ag atoms. The partial DOS of Zn, O and Ag when ${V}_{Zn}$ is far from both doped Ag atoms, have been shown in Fig. 6e. The ferromagnetic state is stable with a coupling energy of 37 meV. In a similar doped configuration and without any vacancy defect, there was anti-ferromagnetic coupling between magnetic centers with coupling energy of 21 meV. The total magnetic moment of the super cell significantly increased. The magnetic moments on doped Ag atoms are found to be 0.3 and 0.29 *μ*_*B*_ while the total magnetic moment of the super cell becomes 3.91 *μ*_*B*_. The significant spin density was found around the neighboring Zn and O atoms of the impurity and vacancy site (Fig. 6f). Figure 6g shows the super cell of Ag-doped with ${V}_{O}$. The partial DOS of Zn, O and Ag have been shown in Fig. 6h. This indicates that the presence of ${V}_{O}$ does not show magnetism in Ag-doped ZnO.

## Discussion

**Figure 7:**
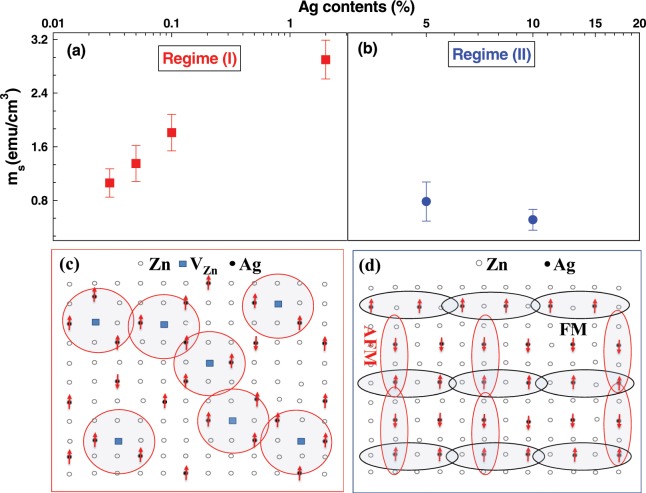
The ${m}_{s}$ per cm^3^ *vs* different Ag contents (%) at 300 K in (**a**) low doping regime (I) and (**b**) higher doping regime (II). (**c**) Schematic representation of long-range ferromagnetic order in Ag-doped ZnO mediated through overlapping of BMPs in regime (I). (**d**) Schematic representation of ferromagnetic Ag^2+^-Ag^2+^ interaction in same Zn plane and anti-ferromagnetic Ag^2+^-Ag^2+^ in different Zn planes in regime (II).

From variety of experimental observations such as Mott VRH, EPR and PL measurements, temperature independent magnetization, and increase of ${m}_{s}$ with ${V}_{Zn}$, it can be emphasized that BMP model is the mechanism for RTFM in Ag-doped ZnO. This has been corroborated by the first principle DFT calculations as discussed before. We have considered two different regimes: low doping regime (I) (0.03%, 0.05%, 0.1% and 2%) and high doping regime (II) (5% and 10%) as shown in Fig. 7a,b. The direct exchange interaction between two Ag^2+^ ions is not possible in regime I as the average distances between two Ag^2+^ ions in 0.03%, 0.05% and 0.1% Ag-doped ZnO *i*.*e*. 2.2 nm, 1.8 nm and 1.5 nm, respectively, are very large with respect to the lattice parameters of wurtzite ZnO. This strongly implies the important role of ${V}_{Zn}$ and ${V}_{O}$ for ferromagnetic interaction between Ag atoms. As revealed from DFT calculations and Fig. 4d, ${V}_{Zn}$ (${V}_{O}$) enhance (destroy) the ferromagnetic order in Ag-doped ZnO. Therefore, the role of ${V}_{Zn}$ in ferromagnetic interaction in regime I can be explained through the interaction between BMPs. These BMPs are formed by the magnetic exchange interaction between spins of Ag^2+^ ions and spin of the localized acceptor hole on ${V}_{Zn}$^23,68,69^. The radius of BMP associated with ${V}_{Zn}$ is, $r=\varepsilon ({m}_{e}/{m}^{\ast }){a}_{\circ }$, where ${a}_{\circ }$, $\varepsilon$, ${m}_{e}$ and ${m}^{\ast }$ are the Bohr’s radius, high-frequency dielectric constant, free electron mass and the effective mass of hole in ZnO *i*.*e*. ${m}^{\ast }=0.45\,{m}_{e}$, respectively^70,71^. $\varepsilon =21$, gives a value of r = 2.4 nm. The average distance between two Ag^2+^ ions in regime I is smaller than the confinement radius of acceptor hole and this indicates that, at least two Ag^2+^ ions are available for magnetic exchange interaction in the vicinity of a hole due to ${V}_{Zn}$. The Hamiltonian for the magnetic exchange interaction among Ag^2+^ ions via localized hole within a BMP is expressed as^68,69^,

6 $$H=j\,\mathop{\sum }\limits_{i=1}^{\infty }\,{s}_{i}.({S}_{i}+{S}_{i+1})$$

where S and s are the spin of Ag^2+^ ion and hole, respectively. J is the exchange interaction between spins of Ag^2+^ through localized hole. As the concentration of ${V}_{Zn}$ or the localized hole increases; neighboring BMPs overlap and interact with each other. Percolation occurs when BMPs fill roughly ~14–15% of the sample area as the packing fraction and ${x}_{p}$ in ZnO are 0.74 and 0.198, respectively^24^. The percolation threshold (${\delta }_{p}$) for long-range ferromagnetic order of BMPs is estimated to be ~5.6 × 10^−5^. Taking the cation density as 3.94 × 10^22^ cm^−3^ in ZnO^23^, the defect concentration (${V}_{Zn}$) has to be ~2 × 10^18^ cm^−3^ for long range order through overlap of BMPs. This value matches well with the ${V}_{Zn}$ concentration calculated from EPR measurement. The BMP concentration can be estimated from the fitting of experimental M-H data with following relation^63^

7 $$M={n}_{B}{m}_{s} {\mathcal L} (x)+{\chi }_{m}H$$

where L(x) is the Langevin function with $x=({m}_{eff}H)/({k}_{B}T)$, ${m}_{eff}$, ${k}_{B}$, ${\chi }_{m}$ and n_*B*_ are an effective spontaneous moment per BMP, the Boltzmann constant, the susceptibility of the matrix and the number of BMPs, respectively. The fitting of M-H curves of the Ag-doped ZnO films is shown in Fig. 4f. The calculated values of BMPs at 0.03%, 0.05% and 0.1% Ag-doped ZnO are greater than ${\delta }_{p}$ (Fig. 4e) required for long-range ferromagnetic order in ZnO at 300 K. The overlapping of BMPs causes the alignment of Ag^2+^ spins, resulting long range FMO along the percolation path at 300 K (Fig. 7c). From the DFT calculations of 2% Ag-doped ZnO, there are three type of magnetic exchange interactions (i) the Ag^2+^-Ag^2+^ interaction in same Zn plane is ferromagnetic with coupling energy of 44 meV, (ii) the Ag^2+^-Ag^2+^ interaction in different Zn planes is anti-ferromagnetic with coupling energy of 21 meV, and (iii) the Ag^2+^-Ag^2+^ interaction in different Zn plane in the presence of ${V}_{Zn}$ is ferromagnetic with coupling energy of 37 meV. As the number of BMPs (or ${V}_{Zn}$) is greater than ${\delta }_{p}$, the Ag^2+^-Ag^2+^ interaction in different Zn plane in the presence of ${V}_{Zn}$ retards the anti-ferromagnetic interaction in 2% Ag-doped ZnO. This is the reason why we obtain maximum value of m_*s*_ per cm^3^ in 2% Ag-doped ZnO. Finally, in regime II (i) Ag impurities start occupying more Zn sites of the different Zn plane, resulting antiferromagnetically aligned spins, and (ii) at higher doping, Ag^2+^ also starts occupying ${V}_{Zn}$ sites resulting decrease in ${V}_{Zn}$ or BMP concentration (Fig. 4e). As Ag concentration increases (Regime II), anti-ferromagnetic coupling due to Ag^2+^-Ag^2+^ dominates and reduces m_*s*_ per cm^3^ to a large extent (Fig. 4e).

## Conclusions

We have investigated RTFM in Ag-doped ZnO at various doping concentration from 0.03% to 10%. The ${m}_{s}$ per Ag^2+^ ion decreases with Ag-content and does not depend on temperature. The observation of large value of ${m}_{s}$ at very low doping concentration which is much below the ${x}_{p}$ of ZnO, strongly implies the role of native defects in FMO. The increase in ${m}_{s}$ with ${V}_{Zn}$ further illustrates the role of defects on RTFM. The presence of defects within Ag-doped ZnO films have been investigated by EPR and PL measurements. The oxidation states of Ag in Ag-doped ZnO have been determined by XPS. The first principle DFT calculations show that Ag^2+^-Ag^2+^ interaction is ferromagnetic in same Zn plane with magnetic coupling energy of 44 meV and anti-ferromagnetic in different Zn planes with magnetic coupling energy of 21 meV. However, the ${V}_{Zn}$ changed the anti-ferromagnetic state to ferromagnetic state with coupling energy of 37 meV whereas ${V}_{O}$ retards the ferromagnetic ordering in Ag-doped ZnO. Finally, we show that the overlapping of BMP responsible for long-range FMO at room temperature. In regime I, the calculated values of BMPs are greater than the ${\delta }_{p}$ resulting long-range ferromagnetic order. Due to less number of BMPs than the ${\delta }_{p}$ in regime II, antiferromagnetic interaction in different Zn planes starts dominating and reduces the value of *m*_*s*_ to a large extent.
